# Next generation sequencing-based expression profiling identifies signatures from benign stromal proliferations that define stromal components of breast cancer

**DOI:** 10.1186/bcr3586

**Published:** 2013-12-17

**Authors:** Xiangqian Guo, Shirley X Zhu, Alayne L Brunner, Matt van de Rijn, Robert B West

**Affiliations:** 1 Department of Pathology, Stanford University School of Medicine, Stanford, CA 94305-5324, USA

## Abstract

**Introduction:**

Multiple studies have shown that the tumor microenvironment (TME) of carcinomas can play an important role in the initiation, progression, and metastasis of cancer. Here we test the hypothesis that specific benign fibrous soft tissue tumor gene expression profiles may represent distinct stromal fibroblastic reaction types that occur in different breast cancers. The discovered stromal profiles could classify breast cancer based on the type of stromal reaction patterns in the TME.

**Methods:**

Next generation sequencing-based gene expression profiling (3SEQ) was performed on formalin fixed, paraffin embedded (FFPE) samples of 10 types of fibrous soft tissue tumors. We determined the extent to which these signatures could identify distinct subsets of breast cancers in four publicly available breast cancer datasets.

**Results:**

A total of 53 fibrous tumors were sequenced by 3SEQ with an average of 29 million reads per sample. Both the gene signatures derived from elastofibroma (EF) and fibroma of tendon sheath (FOTS) demonstrated robust outcome results for survival in the four breast cancer datasets. The breast cancers positive for the EF signature (20-33% of the cohort) demonstrated significantly better outcome for survival. In contrast, the FOTS signature-positive breast cancers (11-35% of the cohort) had a worse outcome.

**Conclusions:**

We defined and validated two new stromal signatures in breast cancer (EF and FOTS), which are significantly associated with prognosis. Our group has previously identified novel cancer stromal gene expression signatures associated with outcome differences in breast cancer by gene expression profiling of three soft tissue tumors, desmoid-type fibromatosis (DTF), solitary fibrous tumor (SFT), and tenosynovial giant cell tumor (TGCT/CSF1), as surrogates for stromal expression patterns. By combining the stromal signatures of EF and FOTS, with our previously identified DTF and TGCT/CSF1 signatures we can now characterize clinically relevant stromal expression profiles in the TME for between 74% to 90% of all breast cancers.

## Introduction

The tumor microenvironment (TME) is comprised of fibroblasts, endothelial cells, immune cells and extracellular matrix (ECM). The cells within the TME have been demonstrated to play significant roles in the development and progression of cancer [1-8], but most studies view each of these cell groups as a relatively uniform population that is similar across different patients.

Profiling the non-neoplastic cells within the TME directly is difficult due to the variety and relative paucity of these cells in the tissue and practical issues with the isolation of these cells. Our approach rests on the hypothesis that, similar to lymphomas where each tumor is a clonal outgrowth of a particular lymphoid cell type, each soft tissue tumor type can also be regarded as a clonal outgrowth of a particular connective tissue cell type to represent subclasses of stromal proliferation that occur in epithelial tumors.

In our approach, soft tissue tumors (STTs) which are a homogenous collection of a single mesenchymal cell type have phenotypes distinct from each other, can be easily profiled and act as “discovery tools” for various types of TME expression patterns to yield a relatively uniform signature. Using gene array-based expression profiling of fresh frozen specimens of fibroblastic tumors (desmoid type fibromatosis-DTF and solitary fibrous tumor-SFT) and macrophage-rich tumors (tenosynovial giant cell tumor-TGCT/CSF1), we previously discovered novel types of stromal reaction patterns that emphasize the variations in the fibroblast and macrophage compartment in breast cancer between different patients [1,2,8,9]. The biological significance of the identification of these stromal reaction patterns was borne out by the fact that several of these stromal expression patterns have prognostic significance independent from traditional prognosticators such as tumor size, tumor grade and even lymph node status [1,2,8].

Based on our previous findings, the DTF signature robustly defined a stromal pattern for 25 to 35% of invasive breast cancers [2], while the TGCT/CSF1 signature was found in 17 to 28% of breast cancers [1]. However, a significant number of breast cancers were not classified by these signatures. In order to find additional stromal patterns we performed gene expression profiling on a spectrum of fibroblastic lesions. As most of these lesions are quite small, they are routinely entirely submitted as formalin fixed paraffin embedded (FFPE) tissue. Here, we have applied an RNA-Seq method for the expression profiling of archival, FFPE tissue, termed 3SEQ (3′end RNA sequencing), which we have previously developed. This method can be used to perform global gene expression profiling of FFPE material [10-12], as well as to discover and characterize expression levels for lncRNAs [13]. Within this study, we have used 3SEQ to determine specific gene expression signatures for 10 types of fibroblastic tumors and found that 2 can identify breast cancers with distinct clinical outcome. Taken together with the two previously identified stromal signatures (DTF and TGCT/CSF1 signatures), the combined four stromal signatures now classify 74% to 90% of breast carcinomas.

## Methods

### Samples selection, treatment and 3SEQ analysis

Paraffin blocks from 53 fibrous tumors were collected from the Department of Pathology at Stanford University Hospital, with Health Insurance Portability and Accountability Act (HIPAA)-compliant Stanford University Medical Center Institutional Review Board approval. And the tissues were collected with a waiver of consent due to the archival nature of the specimens. The samples consisted of collagenous fibroma (FC, six cases), elastofibroma (EF, four cases), infantile digital fibromatosis (IF, three cases), palmar fibromatosis (PF, eight cases), nasopharyngeal angiofibroma (NPAF, six cases), fibroma of tendon sheath (FOTS, four cases), nodular fasciitis (NF, six cases), dermatofibrosarcoma protuberans (DFSP, four cases), desmoid type fibromatosis (DTF, seven cases) and solitary fibrous tumor (SFT, five cases). Multiple 2 mm-diameter cores were taken from diagnostic FFPE material for RNA isolation as reported previously [12].

### 3SEQ sequencing and data analysis

3SEQ libraries for next generation sequencing-based expression profiling were built according to previously described methods [10-13] and protocols in our lab website [14], and then were sent to Stanford Center for Genomics and Personalized Medicine to be sequenced directionally (36 bp) from 5′end of mRNA fragments towards their poly(A) ends using Illumina GA IIx machines (Illumina, Inc., San Diego, CA, USA).

Sequence reads, after filtering for read quality, were re-filtered by fastx (fastx_artifacts_filter) [15], and mapped to the transcriptome (refMrna, downloaded from the UCSC genome browser, http://www.genome.ucsc.edu/) by using SOAP2, allowing at most two mismatches [16]. Total numbers of sequence reads for each gene symbol from the transcriptome mapping were determined and were used to create the gene-expression profile matrix. Read counts from each library were normalized to transcripts per million reads (TPM). Adequacy of sequencing depth for each library was assessed using an estimation of library saturation for transcriptome detection, which showed that our sequencing depths were sufficient to detect an average of 67% of reference mRNAs. The distances between sequencing reads and 3′ ends of mRNAs were measured to be around 100 to 200 nt as we expected based on the length of mRNA after shearing.

Hierarchical clustering was performed as previously described (filter by SD100, adjust data by “log transform data” and “center genes”, then perform Hierarchical clustering with “Spearman Rank Correlation” and “Centroid Linkage”) [8] and a clustered heatmap was visualized with Java TreeView (http://sourceforge.net/projects/jtreeview/files/). Two class SAM analysis (Significance Analysis of Microarrays) [17] was used to identify genes expressed differentially between each type of fibrous tumor versus the other fibrous tumors with FDR of 0.05. Genes that were specific for a particular tumor type and that were highly expressed were used to define the fibrous tumor signature for each tumor type. All gene expression profiling data used for this study have been deposited in the Gene Expression Omnibus (GEO) and are publicly accessible through GSE42948. The 3SEQ and SAM analyses scripts for this study are attached as Additional file 1.

### Analysis of breast cancer microarray expression data

Four publicly available breast cancer expression datasets with clinical follow-up information were used for this study (NKI [18], GSE1456 [19], GSE3494 [20] and GSE4922 [21]). The combined sets of positively expressed genes that are unique for each fibrous tumor signature were identified in each of the four breast cancer datasets. Using only the expression data for these genes, the breast cancers were grouped by unsupervised hierarchical clustering using Cluster 3 software (http://bonsai.hgc.jp/~mdehoon/software/cluster/). The resulting heatmap was visualized with Java TreeView. We then identified the breast cancer cases that were most closely associated with each fibrous tumor signature. These cases were visually identified in the heatmap as sharing the largest group of genes with high expression. These observations were validated by calculating the volume of expression (the averaged expression values of each probe within the gene set after removing all the negative values in the matrix) to confirm that the visually identified group of cases had indeed the highest levels of expression relative to other groups of cases.

After studying 10 fibrous tumor signatures in four breast cancer expression datasets, we identified the fibrous tumor signatures which significantly stratify breast cancers into two groups with different outcomes; only the EF and FOTS signatures divided each of four breast cancer datasets into two groups with statistically significant outcome differences in the four datasets. To refine our analysis, we determined the “core” genes for EF and FOTS signatures: genes that are consistently coordinately and highly expressed in the breast cancer datasets. We defined the EF and FOTS core genes as those exhibiting greater than 0.1 correlated expression and present in at least three of the four gene clusters in four datasets [1,2].

For the association analysis between core gene signatures, a chi-square test was performed using the software GraphPad (GraphPad Software, Inc., La Jolla, CA, USA). Kaplan-Meier plots were used to compute the survival curves, and Log-rank (Mantel-Cox) Test was used to determine the statistical significance of survival between groups by using GraphPad Prism5 software. Univariate and multivariate analysis by the Cox proportional hazard method was performed by using the survival package in R. Analysis of gene ontology (GO) and the Kyoto Encyclopedia of Genes and Genomes (KEGG) Pathway was done by using DAVID Bioinformatics Resources version 6.7 (http://david.abcc.ncifcrf.gov/).

## Results

### Expression of fibrous tumor gene signatures in breast cancer

**Figure 1:**
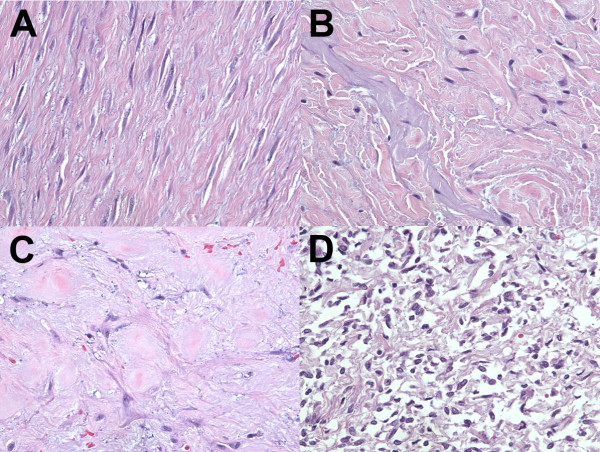
**H&E images of representative fibroblastic tumors profiled by 3seq. A)** Desmoid type fibromatosis; **B)** Elastofibroma; **C)** Fibroma of tendon sheath; **D)** Solitary fibrous tumor.

**Figure 2:**
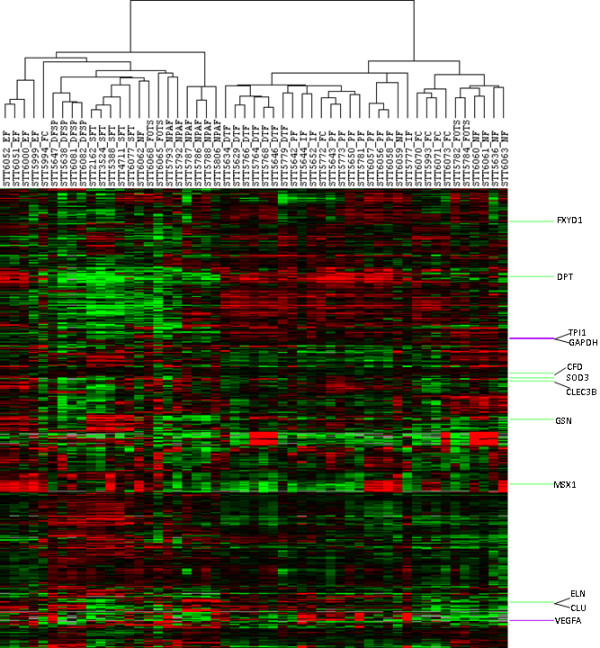
**Unsupervised hierarchical clustering of expression profiles of 10 groups of 53 fibrous tumors.** Unsupervised hierarchical clustering of 53 tumors (columns) using 961 genes (rows). Within the heatmap, red represents higher expression, black represents mean expression, green represents lower expression, and gray represents normalized expressional level zero. Note: In several instance tumors of the same diagnosis appear adjacent to each other in the figure. It should be noted that the arms of the dendrogram can rotate freely and that it is the length of the dendrogram arms that determines the similarity between cases.

Gene expression profiling by 3SEQ was performed on 53 fibrous tumors representing 10 groups of benign fibrous soft tissue tumors (Figures 1 and 2, see Additional files 2, 3, 4). For each diagnostic entity, up to 2,598 genes were highly expressed relative to all other tumors based on two-class SAM analysis, defining 10 unique fibrous tumor gene signatures (Table 1, see Additional file 5).

**Table 1:** Differentially expressed genes between each type of tumors and all others by SAM

	**Diagnosis of fibrous tumors**	**Sigillum**	**Number of cases**	**Number of genes up-expressed**	**Number of genes down-expressed**
1	**Desmoid type fibromatosis**	DTF	7	42	0
2	**Solitary fibrous tumor**	SFT	5	2,598	51
3	**Dermatofibrosarcoma protuberans**	DFSP	4	452	8
4	**Collagenous fibroma**	FC	6	30	0
5	**Elastofibroma**	EF	4	259	279
6	**Infantile digital fibromatosis**	IF	3	191	0
7	**Palmar fibromatosis**	PF	8	166	1
8	**Nasopharyngeal angiofibroma**	NPAF	6	273	26
9	**Fibroma of tendon sheath**	FOTS	4	66	0
10	**Nodular fasciitis**	NF	6	89	0

To the fibrous tumor gene signatures in breast cancer, we analyzed four publicly available breast cancer datasets (GSE1456, GSE4922, GSE3494 and the NKI Dataset). Each breast cancer dataset was clustered separately with the gene sets specific for each of the 10 fibrous tumor signatures. We identified the fibrous tumor genes coordinately over-expressed in a subset of breast cancers for each dataset as representing the stromal expression pattern in the breast cancers that is most similar to that particular fibroblastic tumor. Four fibrous tumor signatures did not show coordinated expression patterns in the four breast cancer datasets. Another four fibrous tumor signatures demonstrated coordinated expression patterns in the breast cancer datasets, but did not consistently show a statistically significant difference in the prognosis between the two groups of breast cancers defined by these fibrous tumor signatures. On the contrary, both the EF and FOTS gene signatures demonstrated meaningful outcome results for survival in all four breast cancer datasets (see Additional file 6).

### Core gene sets of FOTS and EF and their implication in the prognosis of breast cancer

**Figure 3:**
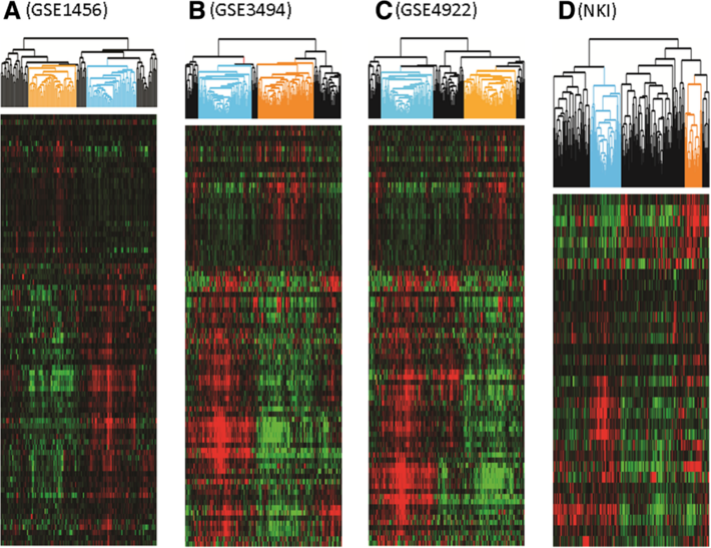
**Hierarchical clustering of four breast cancer datasets by FOTS/EF core gene sets.** Within the heatmap, red represents higher expression levels, green represents lower expression levels, and black represents mean expression levels. Sample clusters designated with Orange and Blue trees are FOTS + breast cancers and EF + breast cancers in GSE1456 **(A)**, GSE3494 **(B)**, GSE4922 **(C)** and NKI dataset **(D)**.

**Figure 4:**
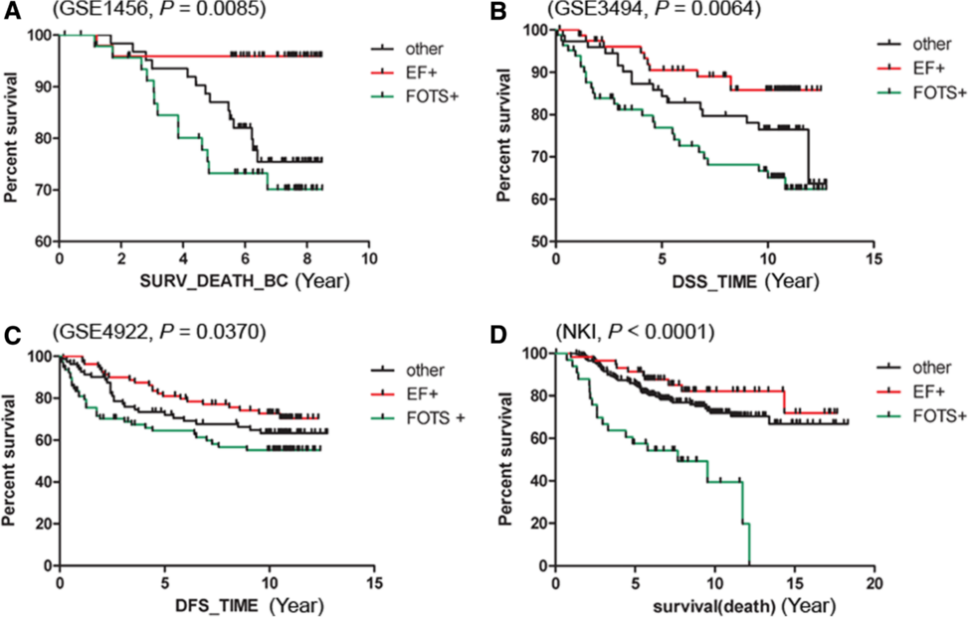
**Kaplan-Meier plot for breast cancer cases stratified by FOTS/EF core gene sets.** The Kaplan-Meier survival curves are tested by the Log-rank (Mantel-Cox) Test to determine the statistical significance of association of EF+, FOTS + or other breast cancers with overall survival (**A**, *P* = 0.0085), disease specific survival (**B**, *P* = 0.0064), disease free survival (**C**, *P* = 0.0370), and overall survival (**D**, *P* <0.0001) within the four breast cancer datasets, respectively.

To extend our analysis, we determined the fibrous tumor “core” gene signature as being the genes coordinately over expressed in all four breast cancer datasets as we previously had defined the core gene set for DTF signature [2], by requiring that each “core” gene has to be present in at least three of the four gene clusters in four datasets. For each core gene signature and breast cancer dataset, the group of breast cancer samples with the highest aggregate of expression of fibrous tumor signature genes was identified as having a correlation >0.2 (Figure 3). The breast cancer subgroups were assessed for clinicopathologic correlations in each of the four breast cancer datasets. The EF core gene signature positive breast cancers demonstrated significantly better outcome (*P* <0.05) for survival than EF core gene signature-negative cases (Figures 3 and 4). EF-like breast cancers account for 20 to 33% of all breast cancers in each cohort (see Additional file 7). The EF signature was defined by 41 genes (see Additional file 8). In contrast to the EF signature, the FOTS core gene signature positive breast cancers had a worse outcome than breast cancers (*P* <0.05) that failed to express it (Figures 3 and 4). FOTS-like breast cancers account for 11 to 35% of all breast cancers in each cohort (see Additional file 7). This gene set was comprised of 16 genes (see Additional file 8). Multivariate analysis showed that both the FOTS and EF signatures are independent of other clinical parameters, such as tumor grade, lymph node status, ER expression and so on (see Additional file 9).

To better understand the functions of the two new stromal core gene set signatures (EF and FOTS), we performed Gene Ontology (GO) and KEGG pathway analysis. The results show that the EF core genes are significantly enriched in biological processes including ‘response to wounding’ and BMP signaling, were enriched in Pathways including Tyrosine metabolism, Complement and coagulation cascades (see Additional file 8). For the FOTS core gene set, biological processes, including glycolysis, were enriched based on the 16 core genes (see Additional file 8), KEGG pathways including “Glycolysis/Gluconeogenesis” and “Fructose and mannose metabolism” were enriched (see Additional file 8).

### Independent signatures of FOTS and EF with DTF fibroblast and TGCT/CSF1 macrophage signatures

Previously, our group reported that the DTF fibroblast signature is associated with good outcome in breast cancer [2,8], and that the TGCT/CSF1 macrophage signature is associated with higher tumor grade in breast cancer [1]. We compared these signatures with those of EF and FOTS to determine the degree of overlap between cases with either of the two good prognosis signatures (EF and DTF fibroblast) and the overlap between cases with either of the bad prognosis signatures (FOTS and TGCT/CSF1 macrophage) in the four independent breast cancer datasets (NKI, GSE1456, GSE3494 and GSE4922). In order to make these signatures more comparable and reproducible, we defined the DTF fibroblast breast cancers and TGCT/CSF1 macrophage breast cancers by the same criteria as the criteria for EF/FOTS-like cases (cases with high expression of signature and >0.2 correlation within clusters). Chi-square test indicated that the association between DTF fibroblast cases and EF-like cases is not statistically significant in three of four datasets although there are overlapping assignments by these two signatures. Likewise, the association between TGCT/CSF1 macrophage cases and FOTS-like cases is also not statistically significant in three of four datasets (see Additional file 10). These data show that the signatures associated with good outcome (DTF and EF) identify non-overlapping sets of tumors and that similarly the two signatures associated with poor outcome (TGCT/CSF1 and FOTS) also describe distinct sets of tumors.

**Figure 5:**
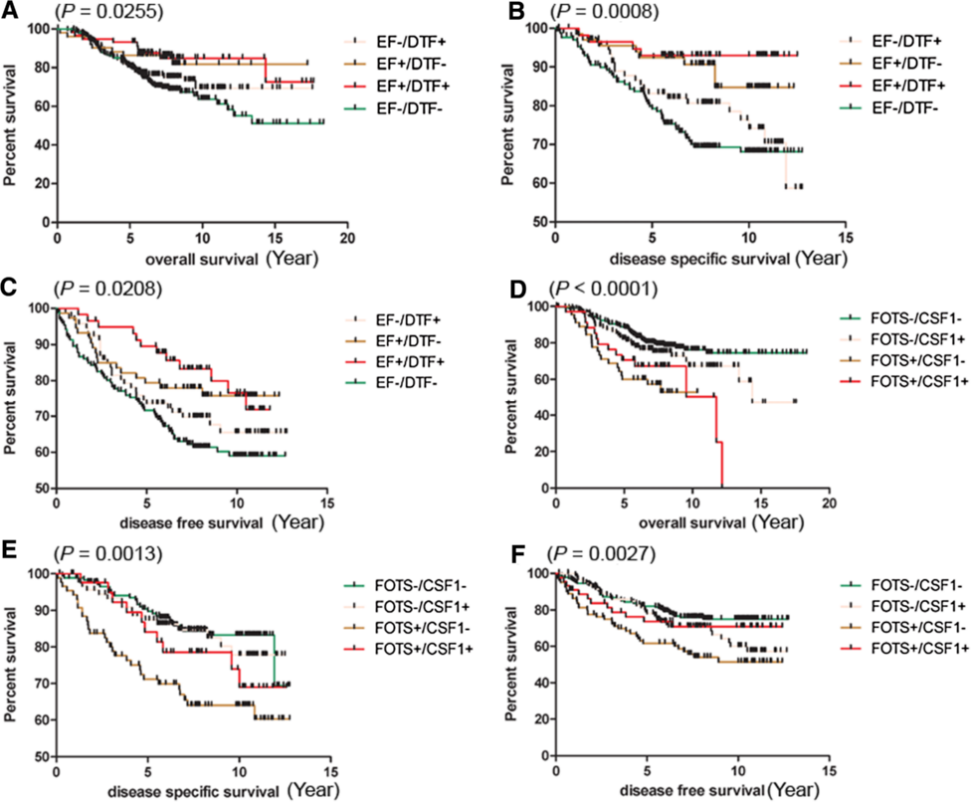
**Kaplan-Meier plot with Log-rank (Mantel-Cox) test for the combined signatures.** The plots and tests were performed between four categories of EF±/DTF ± **(A, B, C)** or FOTS±/TGCT/CSF1 ± **(D, E, F)** breast cancers with overall survival **(A, D)**, disease specific survival **(B, E)**, and disease free survival **(C, F)** (TGCT/CSF1 group is abbreviated to CSF1).

To examine the degree to which the two new signatures provide additional information to the previously identified DTF/TGCT/CSF1 signatures, we stratified breast cancer into four categories based on DTF/EF signatures for good outcome (DTF+/EF+, DTF+/EF-, DTF-/EF+, DTF-/EF-) or FOTS/TGCT/CSF1 signatures for poor outcome (FOTS+/TGCT/CSF1+, FOTS-/TGCT/CSF1+, FOTS+/TGCT/CSF1-, FOTS-/TGCT/CSF1-) in the four breast cancer datasets. EF+/DTF + breast cancers account for 11 to 16% of the cases, while EF-/DTF- breast cancers account for 44 to 47% of the cases. FOTS+/TGCT/CSF1+ breast cancers account for 6 to 12% of the cases, whereas FOTS-/TGCT/CSF1- breast cancers account for 41 to 65% of the cases in the four breast cancer datasets (see Additional file 7). The prognosis of these four categories for DTF/EF signatures or FOTS/TGCT/CSF1 signatures was assessed by overall survival (OS), disease specific survival (DSS), and disease free survival (DFS) by the pooled cases of four datasets (Figure 5). EF-/DTF- breast cancers demonstrated significantly worst outcome in overall survival, disease specific survival and disease free survival. EF + breast cancers showed the best outcome in overall survival, disease specific survival and disease free survival, independent of DTF signature status, while the EF-/DTF + breast cancers showed better outcome than EF-/DTF- breast cancers. FOTS-/TGCT/CSF1- breast cancers demonstrated significantly better outcome in overall survival, disease specific survival and disease free survival. The FOTS+/TGCT/CSF1- breast cancer showed the worst outcome in overall survival, disease specific survival and disease free survival.

Correlation with conventional clinical parameters indicated that FOTS+/TGCT/CSF1+ breast cancers were significantly more likely to be ER negative (52.63%), PR negative (58.62%), Grade 3 (58.11%) and p53 mutant (55.17%) than FOTS-/TGCT/CSF1- breast cancers with ER negative (6.45%), PR negative (12.5%), Grade 3 (9.06%) and p53 mutant (5.24%). These FOTS+/TGCT/CSF1+ breast cancers were also more likely to be Basal subtype (17 in 34), while FOTS-/TGCT/CSF1- breast cancers were more likely to be Luminal A subtype (94 in 270). EF+/DTF + breast cancers were less likely to be ER negative (7%), PR negative (11.76%), Grade 3 (7.61%) and p53 mutant (8.96%) than EF-/DTF- breast cancers that were ER negative (24.51%), PR negative (31.82%), Grade 3 (40.48%) and p53 mutant (32.88%) (see Additional file 11).

## Discussion

Stromal components within the TME are known to be involved in cancer initiation, progression and prognosis [1-4,6,7,9,22-27]. In many studies, the different cellular components of the TME are treated as relatively invariable factors that are assumed to play a similar role in tumor samples from different patients. However, through systematic analysis of breast cancer H&E images with a novel machine learning based method, C-Path, we have recently shown that the morphological features of the tumor stroma vary markedly between tumor samples and that they are not only significantly associated with survival in breast cancer, but that their impact on outcome is even stronger than the features of the epithelial component itself [9].

By clustering breast carcinoma expression profile datasets using only the genes that are specific for distinct fibroblastic tumors, we can observe subsets of cancer that contain different fibroblastic subtypes in the tumor stroma. In contrast, hierarchical clustering that uses all genes in the dataset obtained from an entire tumor specimen usually groups samples together based predominantly on the gene expression pattern in carcinoma cells as these cells often represent the majority of the cells within a tumor and often show the most variation in expression patterns. Thus, their transcript levels represent the strongest signal in the sample. As a result, differences between tumors based on their stromal expression patterns are often not observed in datasets where the entire gene expression profile of the sample is used.

It is difficult to obtain gene expression profiles from normal fibroblast subtypes as in normal and tumor tissue they are typically closely associated with other cell types, such as epithelial cells, and techniques, such as micro-dissection, are laborious. Our approach rests on the hypothesis that, similar to lymphomas where each tumor is a clonal outgrowth of a particular lymphoid cell type, each fibroblast tumor type can be regarded as a clonal outgrowth of a particular connective tissue cell type [28]. Fibroblastic tumors thus represent neoplasms of different normal fibroblast subtypes and differentially express genes typical for various fibroblast functions. Moreover, each fibroblastic tumor type represents a largely homogenous population of cells and can be robustly profiled. By using this approach, our group has previously demonstrated that a specific stromal gene expression pattern, the DTF fibroblast signature, could robustly and reproducibly define a subgroup of breast cancer patients with good prognosis [2,8] and that a second stromal pattern, the TGCT/CSF1 macrophage signature, is associated with breast cancers of a higher tumor grade, with decreased expression of ER/PR, and increased mutations of TP53 [1]. Subsequent studies have shown that these different TME variants can even be identified in cases of pre-invasive ductal carcinoma [6]. These findings indicated that the type of TME can vary between patients and that expression profiles obtained from STTs form a useful tool to distinguish these TME variants.

Our prior studies allowed us to identify distinct TME subtypes in up to 50% of breast cancers. In order to extend our findings we determined the gene expression profile for an additional eight fibroblastic tumors. Previous studies required fresh frozen tumor samples, but for many of the fibroblastic lesions we intended to analyze only FFPE material was available. We therefore used a novel gene expression profiling approach (3SEQ) that uses next generation sequencing of RNA fragments purified by oligo-dT selection from FFPE material (Additional file 12). Applying eight novel fibrous signatures to four publicly available breast cancer expression profiling datasets, we found that three of these signatures were not expressed in the breast cancers in a coordinated manner. Of the five remaining signatures, three did not show differences in outcome analysis. In contrast, the EF and FOTS signatures could stratify the breast cancer samples into two groups, through highly coordinated gene expression with consistent association with outcome in all the four breast cancer datasets. The EF signature positive breast cancers demonstrated good outcome, while the FOTS signature positive breast cancers showed bad outcome. In this study, the SFT signature is significantly associated with worse outcome only in the NKI dataset, but there is no clear pattern in the other three datasets, consistent with our previous findings [2]. The current DTF-(3SEQ) signature, which is similar to the DTF signature previously defined against SFT [2,8], is associated with good outcome in three of the four breast cancer datasets, though the association in this analysis is not statistically significant. The difference in the significances of old and new signatures related to outcome can be explained by the fact that the genes differentially expressed for a particular lesion is to a great extent determined by the other samples in the dataset to which it is compared. The original DTF was determined through a comparison with SFT only while the currently defined DTF-(3SEQ) signature was determined through a comparison with a much larger number of distinct fibrous tumor types. As a result, the current DTF-(3SEQ) signature contains 42 genes from the comparison between DTF and the other nine types of fibrous tumors including SFT, while the original DTF signature contains 237 genes from the comparison between DTF and only one other type of fibrous tumor, SFT.

The EF and the previously identified DTF fibroblast signature both identify good outcome in breast cancer, while the FOTS and the previously identified TGCT/CSF1 macrophage signature both identify bad outcome in breast cancer. In order to explore the relationships between the good or bad signatures, we compared the breast cancer sample assignments between them. The comparison results showed that 11 to 16% of breast cancers were positive for both EF and DTF core gene sets, while 44 to 47% of breast cancers were negative for both, 23 to 33% of breast cancers were EF-/DTF+, and 9 to 20% of breast cancers were EF+/DTF-. In addition, 6 to 12% of breast cancers were positive for both of FOTS and TGCT/CSF1 core gene sets, 41 to 65% of breast cancers were negative for both FOTS and TGCT/CSF1 core gene sets, 5 to 24% of breast cancers were FOTS+/TGCT/CSF1-, and 23 to 24% of breast cancers were FOTS-/TGCT/CSF1+. EF+/DTF + breast cancer cases were more likely to be ER+/PR+, low grade, with less lymph node than EF-/DTF- breast cancers. FOTS+/TGCT/CSF1+ breast cancers are more likely to be ER-/PR-, high grade, base-like breast cancers than FOTS-/TGCT/CSF1- breast cancer cases.

In order to test the prognosis power of the combined core gene sets of EF, FOTS, DTF and TGCT/CSF1, we pooled the outcome data from four breast cancer datasets in overall survival (OS), disease specific survival (DSS) and disease free survival (DFS). Kaplan-Meier analysis for the combined core gene sets in the pooled dataset showed that EF-/DTF- breast cancers were associated with worst outcome in OS, DSS and DFS, while EF + breast cancers, no matter whether they are DTF- or DTF+, were associated with better outcome in OS, DSS and DFS. FOTS-/TGCT/CSF1- breast cancers were associated with better outcome in OS, DSS and DFS, while FOTS+/TGCT/CSF1- breast cancers were associated with worse outcome in OS, DSS and DFS.

To better understand the potential functions of the two new stromal core gene set signatures (EF and FOTS) in breast cancer, Gene Ontology (GO) and KEGG PATHWAY analysis were performed, which show that the 41 EF core genes are significantly enriched in biological processes including ‘response to wounding’ and BMP signaling. Within the 41 EF core genes, almost one fourth of them (9/41), such as *AOC3*, *AOX1*, *C6*, *CFD*, *CFH*, *CLU*, *GSN*, *LYVE1* and *MECOM*, were related to ‘response to wounding’. The association with wound healing has been previously identified in a study of prognostic gene signatures in breast cancer [29]. However, a comparison between the EF gene list and the previously identified gene signatures of the “minimum number core serum response” (CSR) genes [29] necessary for tumor classification, we found that only one gene, *GSN*, was shared between EF core signature and CSR genes (from quiescent samples, in contrast to activated samples). The BMP pathway is well known to modulate cross-talk between stromal cells and epithelial cells [30-32], and three genes (*CHRDL1*, *GREM2* and *MSX1*) involving BMP signaling were comprised of the EF core signature. For the FOTS core gene set, biological processes, including glycolysis, were enriched based on the 16 core genes. This suggests that the increase in glycolysis, which serves a critical role in cancer cell growth and invasion [33], may also influence stromal fibroblast. *FOXM1* is an example of FOTS core genes involved in both of epithelial cancer cells and the cancer associated fibroblasts. By comparing the gene expression between isolated breast cancer-associated fibroblasts (CAFs) and normal mammary fibroblasts (NFs) isolated from the same patient, Mercier *et al*. found that *FOXM1* was up-regulated in CAFs rather than the NFs [34]. *TPI1* gene in FOTS core signature was up-regulated in the CL4 fibroblast, which could supply epithelial cancer cells with pyruvate/lactate as “fuel” to help epithelial cancer cells to escape the anti-angiogenic treatment [35]; therefore, the TPI1 targeted therapy (or FOTS gene targeted therapy) for cancer-associated fibroblast will be an actionable way to control the progression of cancer in conjunction with anti-angiogenic therapy.

## Conclusions

Our study explores the TME space as defined by the fibroblastic component, by using a wide range of fibroblastic tumors as tools for the definition of distinct tumor stromal responses. Our data confirm the significance of the TME in breast cancer behavior and are consistent with our previous observations on the variability of the TME in breast cancer. By using these two novel signatures in addition to two previously identified DTF and TGCT/CSF1 signatures, we could characterize the stromal expression profile for the tumor microenvironment in 74% to 90% of all breast cancers. Through their association with clinical outcome, the genes within the two core gene sets of FOTS and EF indicate that they play a functional role in the progression of breast cancers. In the future, recognition of the distinct TME types in breast cancer could lead to targeted therapy that is specific for the distinct TME-based subtypes of breast cancer.

## Abbreviations

3SEQ: 3′end RNA sequencing; CAFs: Cancer-associated fibroblasts; DFS: Disease free survival; DFSP: Dermatofibrosarcoma protuberans; DSS: Disease specific survival; DTF: Desmoid type fibromatosis; ECM: Extracellular matrix; EF: Elastofibroma; FC: Collagenous fibroma; FFPE: Formalin fixed, paraffin embedded; FOTS: Fibroma of tendon sheath; GO: Gene ontology; IF: Infantile digital fibromatosis; KEGG: Kyoto encyclopedia of genes and genomes; NF: Nodular fasciitis; NPAF: Nasopharyngeal angiofibroma; OS: Overall survival; PF: Palmar fibromatosis; SAM: Significance analysis of microarrays; SFT: Solitary fibrous tumor; STTs: Soft tissue tumors; TGCT: Tenosynovial giant cell tumor; TME: Tumor microenvironment.

## Competing interests

The authors declare that they have no competing interests.

## Authors’ contributions

XG, MVD and RW designed the research and wrote the paper. XG and AB analyzed the data. SXZ performed the experiments. MVD and RW supervised the research. All authors read and approved the final manuscript.

## Supplementary Material

Additional file 1 **The scripts for 3SEQ and SAM analyses.** This file supplies the perl script “3seq.pl” for 3SEQ analysis and R script “3seq.2class.sam.fibrous.option1.r” for SAM analysis. Click here for file

Additional file 2 **Statistics of 3SEQ libraries.** This file summarized the yield of each 3SEQ library including total reads and uniquely mapped reads to transcriptome. Click here for file

Additional file 3 **The files for clustering and heatmap.** This file includes the txt, atr, gtr, jtv and cdt files for clustering and heatmap. Click here for file

Additional file 4 **The estimation of library depth for mRNA detection of two 3SEQ libraries.** This file assesses the depth of 3SEQ sequencing data. Click here for file

Additional file 5 **Differentially expressed genes from SAM analysis.** This file provides genes differentially expressed between each type of fibrous tumor versus the other fibrous tumors, and these genes were identified by Two Class SAM analysis with FDR of 0.05. Click here for file

Additional file 6 **Summary of outcome for 10 fibrous tumor signatures in four breast cancer datasets.** This file provides the *P*-value from Log-rank (Mantel-Cox) Test of survival between the two breast cancer groups stratified by each fibrous tumor signature in four public available breast cancer datasets. Click here for file

Additional file 7 **Components of each dataset for combined core signatures.** This file presents the breast cancer components defined by combined EF/DTF signature and combined FOTS/TGCT/CSF1 signature in four public available breast cancer datasets. Click here for file

Additional file 8 **GO and KEGG results for FOTS/EF core gene signatures.** This file provides the gene lists for FOTS and EF core gene signatures, the GO and KEGG results for both core gene signatures. Click here for file

Additional file 9 **Multivariate results of FOTS/EF core signatures in four breast cancer datasets.** This file provides the *P*-value and hazard ratio from Cox proportional hazard analysis of fibrous tumor signature and other traditional prognostic factors in four public available breast cancer datasets. Click here for file

Additional file 10 **Association analysis between FOTS and TGCT/CSF1 signatures, or between EF and DTF signatures.** This file provides the *P*-value from Chi-square and Fisher’s exact test for the association between the signatures in four public available breast cancer datasets. Click here for file

Additional file 11 **ClinicoPathological characterization of FOTS±/TGCT/CSF1 ± and EF±/DTF ± signatures.** This file provides the clinicopathological characterization of combined signatures including ER, PR, Grade, Lymph node, P53 and so on. Click here for file

Additional file 12 **Additional supplementary results and discussion.** This file provides some supplementary results about distribution of 3SEQ data, IHC results of Keratin, discusses understanding and reproducibility of EF and FOTS signatures, limitation and application of 3SEQ technique. Click here for file
