# Supplementary figures and images for: Next generation sequencing-based expression profiling identifies signatures from benign stromal proliferations that define stromal components of breast cancer
Source: Breast Cancer Res. 2013 Dec 17;15(6):R117. doi: 10.1186/bcr3586 (PMC3978842; doi:10.1186/bcr3586)

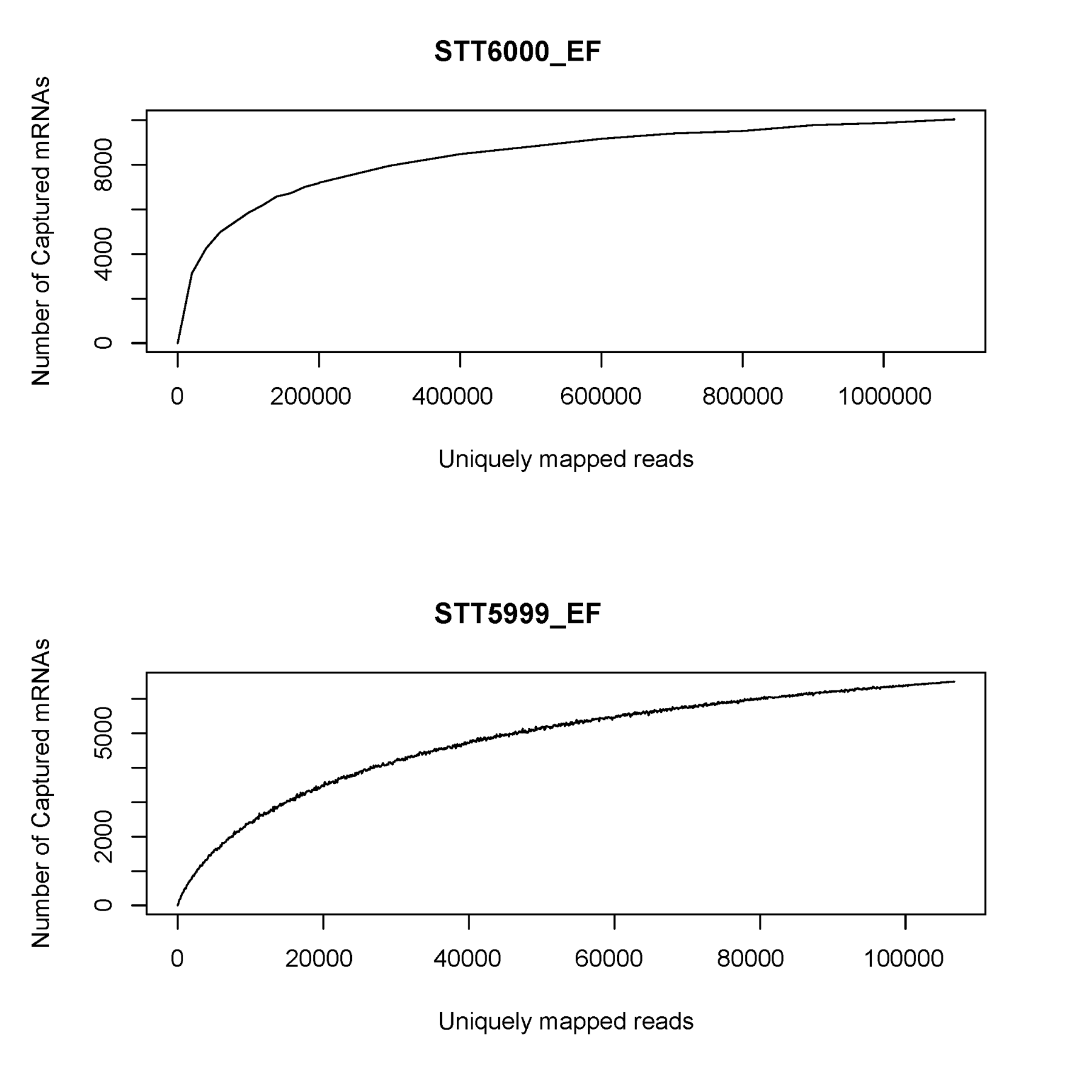

Supplement: Additional file 4 — The estimation of library depth for mRNA detection of two 3SEQ libraries. This file assesses the depth of 3SEQ sequencing data. [file bcr3586-S4.tiff]
