# Supplementary material for: Next generation sequencing-based expression profiling identifies signatures from benign stromal proliferations that define stromal components of breast cancer
Source: Breast Cancer Res. 2013 Dec 17;15(6):R117. doi: 10.1186/bcr3586 (PMC3978842; doi:10.1186/bcr3586)
Supplement: Additional file 12 — Additional supplementary results and discussion. This file provides some supplementary results about distribution of 3SEQ data, IHC results of Keratin, discusses understanding and reproducibility of EF and FOTS signatures, limitation and application of 3SEQ technique. [file bcr3586-S12.docx]

**TABLE OF CONTENTS**

- 1. The normal distribution of 3SEQ expression data after filtering and transformation 2
  2. Independent validation of 3SEQ data by Immunohistochemistry staining (IHC) 2
  3. Understanding EF and FOTS signatures as surrogates for different fibroblast subtypes in the microenvironment of breast carcinoma 3
  4. Constructing the reproducible stromal signatures 4
  5. Limitation and application of 3SEQ technique 5

1. The normal distribution of 3SEQ expression data after filtering and transformation


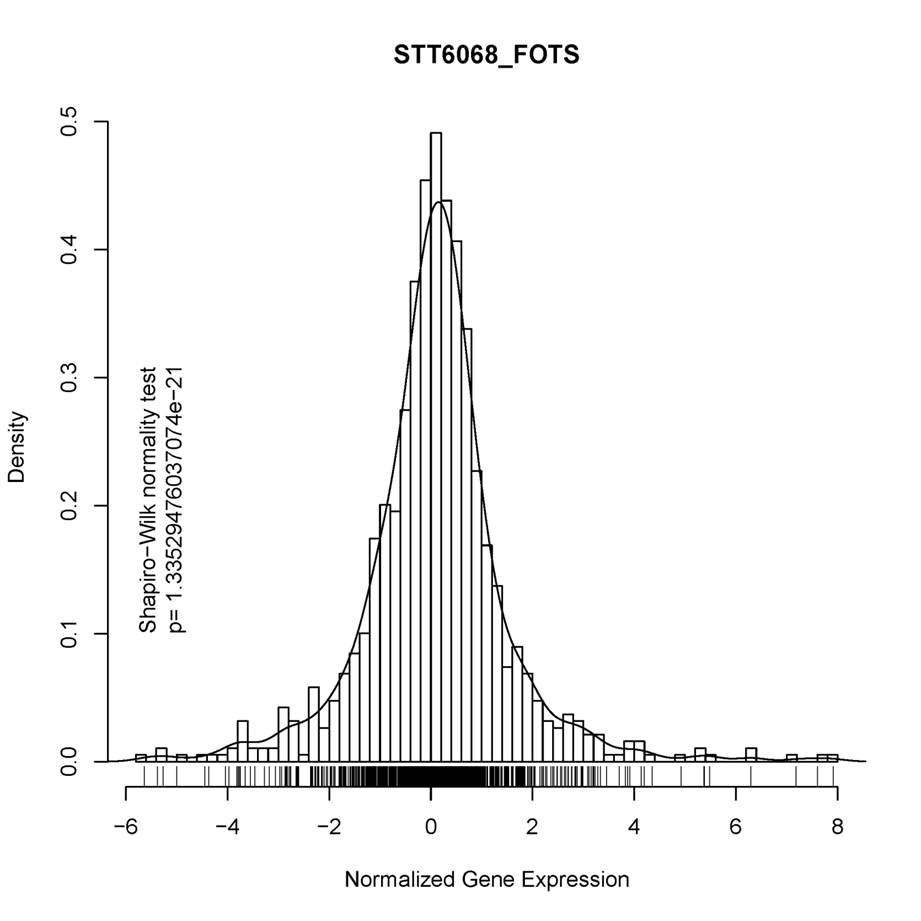


Figure S1: Representative histogram plot of expression profile of 3SEQ and Shapiro-Wilk normality test.

Note. The expression profiles comprised by 961 genes were log transformed and centered by gene as we performed before clustering, and was shown to be normal distribution with p value < 0.01 by Shapiro-Wilk normality test.

2. Independent validation of 3SEQ data by Immunohistochemistry staining (IHC)

The 3SEQ data for IF tumors showed that keratins and other epithelial genes were surprisingly up-regulated in IF tumor. On re-examination of the H&E of the IF cases, it is clear that there are sweat gland elements in these superficial tumor. Analysis of an H&E section of the cores used for expression profiling the 3SEQ approach allows us to see exactly which material went into library construction and the material submitted did indeed contain several sweat glands. In order to validate the expression of these genes in fibrous tumors, we built a fibrous tumor tissue microarray which were comprised of the cases of fibrous tumors analyzed by 3SEQ (6 FC, 4 DFSP, 6 DTF, 4 EF, 2 FOTS, 3 IF, 5 NF, 6 NPAF, 5 PF, 5 SFT). We selected antibodies to 2 epithelial genes found in the 3SEQ expression profile data: anti-CK5/6 and anti-SFN. Immunohistochemistry staining on the microarray showed that both antibodies highlighted the sweat glands within IF, while the other 9 types of fibrous tumors were negative (**Figure S2**). To our knowledge, the finding that sweat glands are associated with IF has not been reported. However, in addition to the “contaminating” sweat glands, we also identified non-epithelial genes in IF, derived from the actual IF lesional cell and these included desmin. Desmin staining demonstrates expression in the IF tumor cells (**Figure S2)**. Importantly, it should be noted that the identification of the gene expression of the associated sweat glands does not affect our breast cancer findings as these genes, being specifically found in IF, are not used in the analysis of EF and FOTS signatures in breast cancer stroma.


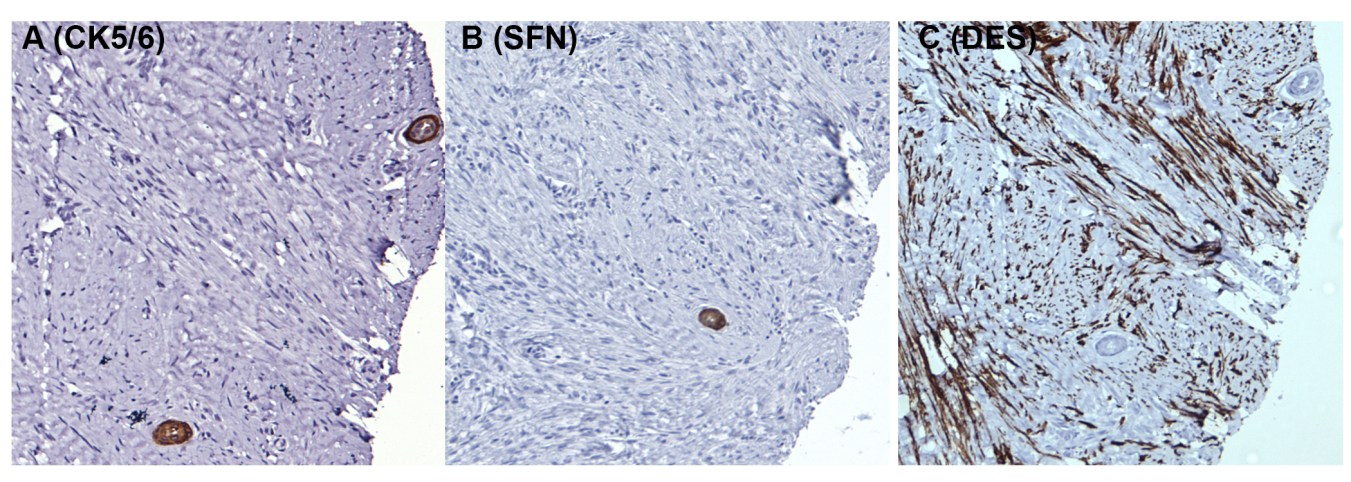


**Figure S2**: IHC staining (Immunohistochemistry staining) of sweat glands in IF with anti-CK5/6 (A) and anti-SFN (B) antibody, of stroma in IF with anti-DES (C) antibody.

3. Understanding EF and FOTS signatures as surrogates for different fibroblast subtypes in the microenvironment of breast carcinoma

Within this study, we need to keep in mind that both FOTS and EF core signatures were originally derived from fibrous tumors that were comprised of homogeneous fibroblast cells, therefore the fibroblast origin of FOTS and EF core signatures could determine that they can represent the expression patterns of fibroblast cells in breast carcinoma, and can be as surrogates to help understand the different fibroblast subtypes in the microenvironment of breast carcinoma.

FOTS core signature is comprised of 16 genes, which were enriched in KEGG pathway including glycolysis, which indicated that FOTS core signature may mimick the “glycolytic phenotype” of cancer-associated fibroblasts in breast cancer (Migneco, Whitaker-Menezes et al. 2010). Besides FOTS signature, 41 EF core genes are significantly enriched in biological processes including ‘response to wounding’ and BMP signaling. These indicated that these molecular expression patterns of EF and FOTS signatures including wound healing, BMP signaling pathway and glycolysis pathway, demonstrated the roles of relevant fibroblast cells in the stroma of solid tumors, and will help to further understand the existence of the EF or FOTS like fibroblasts in the stroma of solid tumor. In addition to glycolysis genes in FOTS signature, hypoxia genes including TPI1/VEGFA/CA9/PFKP were also present in FOTS core signature, this diverse components of FOTS core signature maybe suggest the cross-talk between different cellular processes and the multidimensional variation and complex genetic/physiological factors in the FOTS-like fibroblast of breast cancers (Chang, Sneddon et al. 2004), so as the CSR signature (Chang, Sneddon et al. 2004).

FOXM1 is one of the FOTS core genes, and expressed in proliferating cells including breast cancer–associated fibroblasts. By comparing the gene expression between isolated Breast cancer-associated fibroblasts (CAFs) and normal mammary fibroblasts (NFs) isolated from the same patient, Mercier et al. found that FOXM1 was up-regulated in cancer-associated fibroblasts (CAFs) rather than the normal fibroblasts (NFs) (Mercier, Casimiro et al. 2008).

4. Constructing the reproducible stromal signatures

We know that there are some signatures which lost trace along with time or along with switching lab. Within our study, in order to get the solid EF and FOTS signatures, we tested both signatures in four different breast cancer datasets (NKI, GSE1456, GSE3494 and GSE4922), and found that both signatures were reproducible significantly associated with clinical outcomes.

In addition, we also tested the DTF signature defined previously by papers (West, Nuyten et al. 2005; Beck, Espinosa et al. 2008) in our current study, and showed that DTF signature is significantly associated with good outcome in four breast cancer datasets as we reported before. In order to determine how DTF signature defined by gene microarray study is re-present by 3SEQ study, we performed SAM analysis between only DTF and SFT on 3SEQ data, and showed that 59 of 63 DTF signature genes were also significantly over-expressed in DTF than SFT by 3SEQ data, of 94% DTF signature genes were captured by 3SEQ data again.

5. Limitation and application of 3SEQ technique

For a number of cases in this study, the uniquely mapped reads represent less than 10% of the total reads. This is due to the nature of the starting material. Many of these cases are from old archival material (formalin fixed paraffin embedded, FFPE) and the represented tumors are of low cellularity. In our experience, both these factors contribute to poor RNA quality, leading to many 3SEQ reads of just the poly(A) tail or a significant portion of the poly(A) tail and reducing the percentage of unique reads. However, despite the low percentage of the uniquely mapped reads, we can still generate a robust gene expression profile and overcome the poor percentage of unique reads by sequencing deeply. The resulting total number of unique reads (mean of 1.7 million uniquely mapped reads for the above listed cases with less than 10% reads) is comparable (and on average greater) to our earlier data published in our 2010 paper initially describing the method (Beck, Weng et al. 2010), where we found that the 3SEQ method on FFPE material outperformed conventional microarray methods on case-matched fresh frozen material.

To further analyze the nature of the quality-filtered reads from our set of fibrous tumors, we split all reads into 4 categories: repetitive reads identified by fastx, multiply mapped reads, uniquely mapped reads, and unmapped reads (**Additional file 2**). Repetitive reads, that are mainly poly(A) reads, account for between 10% and 64% of the total reads. For elastofibroma (EF), repetitive reads are between 27% and 33%. Again, the reason why there is a high ratio of poly(A) in the 3SEQ result is that these cases are from FFPE blocks that are old, dating back to1995, and the total RNAs within these blocks is highly degraded. As a result, the poly(A) tail is sequenced if the RNA is highly fragments and many RNA fragments contain only poly(A) RNA after oligo(dT) selection.

Nevertheless, the total number of reads obtained with these older cases is still quite adequate to generate a robust expression profile if we sequence deeply. We can assess the adequacy of sequencing by examining the number of different reference mRNAs (refMrna) detected by 3SEQ with the uniquely mapped reads for each case. As **Additional file 4** shows, although there are less reads and less detected genes for STT5999_EF library (a case with 1% reads uniquely mapped after filtering), we achieve a plateau of different reference mRNAs detected for this library at around 100,000 uniquely mapped reads, similar to the results achieved with STT6000_EF, a case with 8% reads uniquely mapped after filtering.

In conclusion, 3SEQ is a robust technique to do expression profiling for RNAs isolated from both FFPE and fresh frozen materials. For some really old FFPE materials, increasing the sequencing depth will definitely be helpful to increase the sensitivity of expression profiling for some lowly expressed genes.
